# Prognostic Implications of Preoperative Pneumonia for Geriatric Patients Undergoing Hip Fracture Surgery or Arthroplasty

**DOI:** 10.1111/os.12830

**Published:** 2020-10-28

**Authors:** Jia‐wei Shen, Pei‐xun Zhang, You‐zhong An, Bao‐guo Jiang

**Affiliations:** ^1^ Department of Critical Care Medicine Peking University People's Hospital Beijing China; ^2^ Department of Orthopaedics and Traumatology Peking University People's Hospital Beijing China

**Keywords:** Arthroplasty, Hip fracture, Pneumonia, Survival analysis

## Abstract

**Objective:**

To report outcomes of geriatric patients undergoing hip fracture surgery or arthroplasty with or without preoperative pneumonia and to evaluate the influence of pneumonia severity on patient prognosis.

**Methods:**

In this single center retrospective study, we included geriatric patients (≥60 years old) who had undergone hip fracture surgery or arthroplasty at Peking University People's Hospital from January 2008 to September 2018. Patients with fractures caused by neoplasms or patients with incomplete clinical data were excluded. Using logistic regression and the CURB‐65 (confusion, uremia, respiratory rate, blood pressure, and age ≥65 years) score as a prediction tool of 1‐year mortality, the effect of preoperative pneumonia on 1‐year mortality was evaluated. Survival of patients with different response to pneumonia‐specific therapy and survival of patients with different pneumonia severity (evaluated with CURB‐65 score) were analyzed using Cox regression.

**Results:**

A total of 1386 patients were included; among them, 109 patients (7.86%) were diagnosed with preoperative pneumonia. Outcomes were evaluated in August 2019 (at least 1 year after surgery for all patients). Compared to patients without preoperative pneumonia, patients with this condition had higher 30‐day mortality (11.9% *vs* 5%, *P* = 0.002) and 1‐year mortality rates (33.9% *vs* 16.3%, *P* < 0.001) and higher incidence of acute heart failure (7.3% *vs* 3.4%, *P* = 0.034) and acute kidney injury (5.5% *vs* 1.8%, *P* = 0.009). In multivariate regression, preoperative pneumonia was identified as an independent predictor of 1‐year mortality (odds ratio [*OR*], 1.45; 95% confidence interval [*CI*] 1.39–3.52; *P* = 0.021), with other factors including age (≥84 years, *OR*, 1.46; 95% *CI* 1.08–1.60; *P* = 0.027), body mass index (<18.5 kg/m^2^, *OR* 2.23; 95% *CI* 1.52–3.17, *P* < 0.001), anesthesia type (regional, *OR* 0.87; 95% *CI* 0.19–0.97, *P* = 0.042), preoperative pneumonia (*OR* 1.45; 95% *CI* 1.39–3.52; *P* = 0.002), congestive heart failure (*OR* 2.05, 95% *CI* 1.57–6.21, *P* < 0.001), chronic kidney disease (*OR* 1.73; 95% *CI* 1.50–2.62; *P* < 0.001). There was a trend of increased 1‐year mortality as the CURB‐65 score elevated (*P* for trend = 0.006). Cox regression reveals a higher risk of mortality in patient with preoperative pneumonia, especially in patients with no radiologic improvements after therapy (log‐rank, *P* = 0.035). Analysis of the impact of pneumonia severity on patient survival using Cox regression reveals that a CURB‐65 score ≥3 indicated a lower rate of survival (CURB‐65 score of 3: hazard ratio [*HR*] 3.12, 95% *CI* 1.39–7.03, *P* = 0.006; score of 4: *HR* 3.41, 95% *CI* 1.69–6.92, *P* = 0.001; score of 5: *HR* 6.28, 95% *CI* 2.95–13.35, *P* < 0.001).

**Conclusion:**

In this single center retrospective study, preoperative pneumonia was identified as an independent risk factor of 1‐year mortality in geriatric patients undergoing hip fracture surgery or arthroplasty. A CURB‐65 score ≥3 indicated a higher risk of mortality.

## Graphical abstract

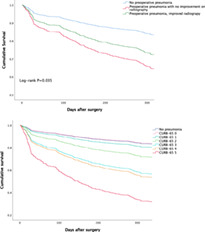

Preoperative pneumonia is one of the underlying comorbidities of geriatric patients undergoing hip fracture surgery or arthroplasty, and has been reported to increase 30‐day mortality rates. However, the influences of preoperative pneumonia and different severities of preoperative pneumonia on long‐term survival have not been reported. As depicted in the graphical abstract, in survival analysis with COX regression, we identified preoperative pneumonia as an independent risk factor of 1‐year mortality in geriatric patients undergoing hip fracture surgery or arthroplasty (Chart A). Patients with unimproved pneumonia after antibiotic and other adjunctive therapies had the lowest rate of survival. When using the CURB‐65 score to evaluate the severity of preoperative pneumonia, only patients with CURB‐65 score ≥3 had a higher risk of mortality, indicating that classification of severity using this scoring system may help to predict the prognosis of patients with preoperative pneumonia.

## Introduction

As the population ages, the incidence of hip fracture is expected to increase worldwide. The incidence each year is expected to rise to 6.26 million by 2050^1^. For geriatric patients, hip fractures often result from underlying osteoporosis and low‐energy injuries^2^; these patients are often frail and suffering from various impairments (e.g. sarcopenia, low physical activity, cognitive decline, depression, and reduced production of hormones, including testosterone, estrogens, insulin‐like growth factor‐1, growth hormone, vitamin D, and pro‐inflammatory cytokines)^3^. Hip fractures greatly influence the quality of life and the survival of patients^4^.

The associated mortality and functional disability after geriatric hip fracture are a huge burden on society^5^, ^6^. Various comorbidities^7^ (e.g. congestive heart failure, myocardial infarction, stroke, diabetes, chronic kidney disease, and preoperative pneumonia) and complications^8^ (e.g. surgical or implant‐related complications, deep venous thrombosis, pulmonary embolism, delirium, postoperative pneumonia, urinary tract infection, acute myocardial infraction, acute heart failure, and acute kidney injury) have been reported to be related to increased 30‐day and 1‐year mortalities^9^, ^10^ following hip fractures.

Among all the complications, pneumonia occurs frequently and may increase the mortality rate by up to four times^10^. Patients with pneumonia may present with coughing, sputum production, dyspnea, or fever^11^. Although great advances have been made in the realm of antibiotic development, pneumonia‐caused mortality is still high as the population of high‐risk patients has increased^12^. In one retrospective study of 467 patients, pneumonia contributed to the highest proportion of causes of 30‐day mortality after hip fracture surgery^13^. Ho *et al*. reported that patients with pneumonia after hip fracture surgery had a lower survival rate 1‐year after surgery (hazard ratio [*HR*] 4.26; 95% confidence interval [*CI*] 1.95–9.31). In society guidelines, prevention and management of postoperative pneumonia has been indicated as crucial in improving patient prognosis^14^, ^15^. A specially designed rehabilitation program after hip fracture surgery can prevent postoperative pneumonia and improve patient outcome^16^.

Preoperative pneumonia has also been reported as an independent risk factor for multiple postoperative complications and poor prognoses in various kinds of surgical patients. Ahsan *et al*. reported that patients with preoperative pneumonia had the highest risk of postoperative respiratory failure^17^. Sarah *et al*. reported an increase in the postoperative mortality rate in patients undergoing orthopaedic, thoracic, and vascular surgeries with preoperative pneumonia^18^. In a large multicenter retrospective cohort study that included 427,656 patients admitted for major general surgeries, preoperative pneumonia was identified as a risk factor of venous thrombosis after surgery^19^. Although preoperative pneumonia was related to poor prognosis after surgery, many patients were still undergoing surgeries when antibiotic therapy was initiated; this may be influenced by the timing of the specific surgery^18^, ^19^. However, unlike postoperative pneumonia, preoperative pneumonia has rarely been a focus in hip fracture studies. Previous studies have reported an incidence of 0.3%–3.2%^20^, ^21^, ^22^, ^23^ for preoperative pneumonia in patients undergoing hip fracture surgery or arthroplasty. In a retrospective study with data from the public health system, Patterson *et al*. reported that preoperative pneumonia was an independent risk factor for 30‐day mortality and surgical‐related complications (including thromboembolic events, myocardial infarctions, and renal insufficiency) in patients with hip fractures, but the disease severity and its influence on long‐term survival, and the influence that pneumonia‐related therapy had on survival were not discussed^23^. There is no study focused on whether improvements on chest radiography after pneumonia‐related treatment in hip fracture patients improves survival. In addition, the diagnostic criteria for preoperative pneumonia in aforementioned studies were not clearly stated, making the interpretation of study results difficult.

The CURB‐65 (confusion, uremia >7 mmol/L, respiratory rate ≥30/min, systemic blood pressure <90 mm Hg, age ≥65 years) score is a validated scoring system for prognostic evaluation for patients with community‐acquired pneumonia. It effectively stratifies patients with different risks of 30‐day mortality and can help clinicians to decide whether a patient needs intensive care monitoring, with a score 0–1 suitable for outpatient care and patients with higher scores more suitable for intensive care^24^. It has been demonstrated in various populations that CURB‐65 is a prediction tool with high efficacy and feasibility. It could be used as a real‐time, electronic decision support tool at the time of patient admission to hospital^25^ and it works better than other scoring systems in predicting hospital mortality in patients with pneumonia^26^, making it a potential candidate for prognosis evaluation for hip fracture patients with preoperative pneumonia. However, no previous studies have assessed the severity of pneumonia in this population using the CURB‐65 scoring system.

Thus, the main purposes of this retrospective study are: (i) to evaluate the clinical outcomes of patients admitted for hip fracture or arthroplasty and diagnosed with or without preoperative pneumonia; (ii) to compare the difference in patient survival between patients with or without improvements on chest radiography after pneumonia‐related treatment; and (iii) to analyze the influence of the severity of preoperative pneumonia (assessed by CURB‐65 score) on long‐term survival in patients undergoing hip fracture surgery or arthroplasties.

## Methods

### *Patients*

All data for patients who were admitted to Peking University People's Hospital between January 2008 and September 2018 were extracted from an electronic medical record system and reviewed.

Inclusion criteria were as follows: (i) geriatric patients (age ≥60 years old); (ii) patients undergoing hip fracture or arthroplasty; (iii) patients with or without preoperative pneumonia; (iv) the study evaluated outcomes including 30‐day mortality, 1‐year mortality, hospital length of stay, intensive care unit (ICU) length of stay, readmission within 1 year, and complications; and (v) a retrospective method.

We exclude patients with: (i) fractures caused by neoplasms; and (ii) incomplete clinical data (e.g. laboratory tests and chest radiographies).

This non‐interventional retrospective cohort study was approved by the Ethics Committee of Peking University People's Hospital (Approval Number: H18REA‐013).

### *Diagnosis, Evaluation, and Therapies for Preoperative Pneumonia*

All patients received a chest X‐ray or chest CT on admission. Patients with pneumonia were treated with antibiotics and other adjunctive therapies, and their chest radiographies were monitored before surgery. Generally, surgery was postponed if patients had fever or hypoxemia (evaluated with pulse oximetry or arterial blood gas analysis), or were supported by mechanical ventilation. Initiation of surgery was at the discretion of the surgeons and pulmonary physicians.

In the data‐reviewing period of our study, two experienced clinicians reviewed all included patients’ chest radiography separately. According to society guidelines^20^, ^21^, a diagnosis of pneumonia requires meeting the following two criteria:

1. Symptoms and signs (e.g. cough, secretion of sputum, fever, and rapid shallow breathing) of an acute lower respiratory tract infection.
2. Chest X‐ray showing airspace opacity, lobar consolidation, or interstitial opacities not due to any other cause (such as pulmonary edema or infarction).

In case of disagreement between reviewers, data and image would be transferred to a senior pulmonology expert for the final decision on the diagnosis of pneumonia. Evaluation of the severity of pneumonia was performed simultaneously with CURB‐65 scoring^15^. The score ranges from 0 to 5, with 1 point for each of the following criteria: (i) confusion; (ii) urea >7 mmol/L; (iii) respiratory rate ≥30/min; (iv) low systolic (<90 mm Hg) or diastolic (≤60 mm Hg) blood pressure; and (v) age ≥65 years.

On reviewing the rechecked chest radiographs for patients with preoperative pneumonia, an improved chest radiograph was defined as areas of airspace opacity, lobar consolidation, or interstitial opacities having decreased or disappeared.

### *Surgery*

Generally, the type of surgery performed was decided according to the protocols of our institution, summarized as follows:

1. For patients with nondisplaced femoral neck fractures, choose internal fixation with cancellous screws or a sliding hip screw (vertical fracture or fracture at the base of femoral neck); choose arthroplasty (hemiarthroplasty if the patient is cognitively impaired or ambulation restricted; total arthroplasty in patients with no cognitive impairment or ambulation restrictions) or sliding hip screw (no comorbidity and no ambulation restriction) when the fracture is displaced.
2. For patients with intertrochanteric fractures, if the fracture is stable, choose internal fixation with sliding hip screw; if it is unstable, use an intramedullary nail.
3. For patients with subtrochanteric fractures, perform internal fixation with an intramedullary nail.

### *Data Collection and Outcome Evaluation*

The baseline data were recorded at the time of admission, including: (i) demographic characteristics (age, sex, and body mass index [BMI]); (ii) evaluation of overall status of patients (American Society of Anesthesiologists physical status classification [ASA score]^18^ and activities of daily living [ADL] score)^19^; (iii) laboratory test results (e.g. for white blood cell count and C‐reactive protein); and (iv) comorbidities and other histories.

Data associated with surgical intervention and some outcomes were recorded at the time of discharge, including: (i) surgical data, such as location of fracture, prefracture walking ability, mechanism of injury, time interval (hours) between admission and surgery, implant type, surgery type (internal fixation or hemi/total‐hip arthroplasty), and anesthesia type (general or regional anesthesia); (ii) characteristics of patients with preoperative pneumonia treatments of antibiotics, antiviral therapy, and adjunctive therapies, and changes on chest radiographies; and (iii) outcomes were evaluated at the time our study was initiated (on 10 August 2019, calculated from the day of surgery), by following up through the medical record system and phone calls. Details of the outcome variables are listed in what follows.

### *Outcome Variables*

#### *Hospital Length of Stay*

Hospital length of stay represnts the duration of a single episode of hospitalization, calculated by subtracting the day of admission from the day of discharge (or death). Hospital length of stay was used to evaluate the efficiency of disease‐related therapy. The advantage of using length of stay is that the status of patient survival did not influence the efficacy of the estimation. A longer hospital stay could result from a more severe or complicated disease status.

#### *Intensive Care Unit Length of Stay*

The ICU length of stay is the number of calendar days a patient stayed in the ICU. ICU length of stay was used to evaluate the efficiency of disease‐related therapy. Similarly to hospital length of stay, patient survival did not influence the efficacy of the estimation. A longer ICU length of stay could result from a more severe or complicated disease status.

#### *30‐day Mortality*

The 30‐day mortality rates were calculated by the number of death cases within each group (i.e. with or without preoperative pneumonia) divided by the number of patients in each group within 30 days after surgery. The mortality rates can be influenced by the medical care, disease status, and comorbidities of patients, or by related therapies.

#### *1‐year Mortality*

The 1‐year mortality rates were calculated by the number of death cases divided by the number of patients in each group within 1 year after surgery. The mortality rates can be influenced by the medical care, disease status, and comorbidities of patients, or by medical or surgical therapies.

#### *Readmission within 1 Year*

Rate of readmission within 1 year was calculated by the number of readmissions into our department for surgical or implant‐related complications (e.g. periprosthetic fracture, hip instability or dislocation, implant failure, and surgical site infection) after discharge from hospital. In this study, this outcome can help to evaluate the quality and efficiency of the surgical procedures as influenced by preoperative pneumonia.

#### *Complications*

Complications recorded after surgical procedures can be surgery‐related or implant‐related (e.g. periprosthetic fracture, hip instability or dislocation, implant failure, and surgical site infection) or hip fracture‐related (e.g. deep venous thrombosis, pulmonary embolism, and delirium) and other complications (e.g. postoperative pneumonia, urinary tract infection, acute myocardial infraction, acute heart failure, and acute kidney injury). In this study, we use this outcome to evaluate the influence of preoperative pneumonia on included patients.

### *Statistical Analysis*

Collected data were recorded in a database, which were subsequently analyzed with SPSS (Version 25, SPSS, Armonk, NY, USA). Comparison of two groups of continuous variables was achieved with the Mann–Whitney *U*‐test. Comparison of categorical variables was performed using the χ^2^‐test or Fisher's exact test as appropriate. Univariate and multivariate logistic models were used to investigate potential risk factors for 1‐year mortality in the study cohort. Stratified analysis of odd ratios of 1‐year mortality by CURB‐65 scores was performed with logistic regression (adjusted for potential risk factors of 1‐year mortality); meanwhile, the *P*‐value for trend was tested by using CURB‐65 as a continuous variable in the logistic regression to test the linear trend between CURB‐65 and 1‐year mortality. A Cox regression curve was constructed to analyze the survival rate of patients without or with preoperative pneumonia (radiologically improved or not), as well as to analyze the difference in the survival rate of patients without preoperative pneumonia and with different pneumonia severity (with CURB‐65). All tests were two‐sided and *P* < 0.05 was considered statistically significant.

## Results

### *Patients*

During the study period, the data of 1398 patients who met our inclusion criteria were collected from medical records; 12 patients were excluded from the study (including 5 patients with fractures caused by neoplasm and 7 patients with incomplete clinical data). Among the included 1386 patients, 109 (7.86%) were diagnosed with preoperative pneumonia by retrospective analysis of medical records and radiological images; 1277 patients were free of preoperative pneumonia. The baseline characteristics of patients with or without preoperative pneumonia are depicted in Table 1. Most of the variables did not differ much between these two groups, except for ADL scores, white blood cells (WBC), C‐reactive protein (CRP) levels, and prefracture walking ability. There were more patients with ADL scores lower than 45 among patients with preoperative pneumonia than in the other group (72.5% *vs* 44.8%). Patients with preoperative pneumonia had higher WBC (12.10 ± 2.36 *vs* 9.05 ± 3.22, *P* < 0.001) and CRP levels (20.46 ± 9.11 *vs* 12.58 ± 5.45, *P* < 0.001).

**TABLE 1:** Baseline characteristics of enrolled patients (number [%] or mean ± SD)

Indexes	No preoperative pneumonia (*n* = 1277)	Preoperative pneumonia (*n* = 109)	*P*
Age (years)			0.482
60–70	363 (28.4)	28 (25.7)	
70–84	568 (44.5)	55 (50.6)	
>84	346 (27.1)	26 (23.7)	
Sex			0.413
Female	829 (64.9)	75 (68.8)	
male	448 (35.1)	34 (31.2)	
Admission BMI (kg/m^2^)			0.335
<18.5	323 (25.3)	36 (33.1)	
18.5–24.9	611 (47.8)	48 (44.0)	
25–29.9	320 (25.1)	24 (21.9)	
≥30	23 (1.8)	1 (0.9)	
ASA score			0.349
1	46 (3.6)	5 (4.6)	
2	642 (50.3)	45 (41.3)	
3	492 (38.5)	49 (45.2)	
≥4	97 (7.6)	10 (0.5)	
ADL score			<0.001
0–20	0 (0.0)	6 (5.5)	
25–40	572 (44.8)	73 (67.0)	
45–60	380 (29.8)	24 (22.0)	
65–80	241 (18.9)	6 (5.5)	
85–100	84 (6.6)	0 (0.0)	
WBC (×10^9^/L)	9.05 ± 3.22	12.10 ± 2.36	<0.001
C‐reactive protein (mg/L)	12.58 ± 5.45	20.46 ± 9.11	<0.001
**Comorbidities**			
Congestive heart failure	181 (14.2)	16 (14.7)	0.580
Prior myocardial infarction	50 (3.9)	5 (4.2)	0.220
COPD	262 (20.5)	21 (19.2)	0.433
Stroke	52 (4.1)	4 (3.8)	0.507
Dementia	253 (19.8)	23 (21.1)	0.251
Diabetes	232 (18.2)	21 (19.4)	0.063
Chronic kidney disease	68 (5.3)	5 (4.8)	0.328
**Surgical characteristics**			
Fracture type			0.201
Femoral neck fracture	614 (48.1)	52 (47.5)	
Intertrochanteric fracture	577 (45.2)	48 (43.6)	
Subtrochanteric fracture	59 (4.6)	4 (3.9)	
Multiple locations	27 (2.1)	5 (5.0)	
Prefracture walking ability			0.070
Independent	741 (58.0)	55 (50.5)	
Crutch or stick	347 (27.2)	29 (26.6)	
Wheel chair	189 (14.8)	25 (22.9)	
Mechanism of injury			0.165
Fall	1007 (78.8)	83 (76.1)	
Road traffic accidents	72 (5.6)	6 (5.5)	
Unknown	148 (11.6)	20 (18.4)	
Surgical delay (h)			0.132
≤24	95 (7.4)	14 (12.8)	
>24 and ≤48	202 (15.8)	16 (14.7)	
>48	980 (76.7)	79 (72.5)	
Implant type			0.204
Cancellous screw	356 (27.8)	39 (35.8)	
Sliding hip screw	360 (28.2)	23 (21.1)	
Hemiarthroplasty	255 (20.0)	25 (22.9)	
Total hip arthroplasty	237 (18.6)	15 (13.8)	
Intramedullary nail	69 (5.4)	7 (6.4)	
Anesthesia type			0.808
General	257 (20.1)	23 (21.1)	
Regional^*^	1020 (79.9)	86 (78.9)	

^*^ Epidural, spinal anesthesia, or peripheral neuro blockade.

Values were given as number (percentage) or mean ± standard deviations.

ADL, activities of daily living; ASA, The American Society of Anesthesiologist; BMI, body mass index; COPD, chronic obstructive pulmonary disease; WBC, white blood cell count.

### *Characteristics of Treatments for Preoperative Pneumonia*

As described in Table 2, patients identified as having preoperative pneumonia all received antibiotic therapy on admission. Fluroquinolones or beta‐lactams were most frequently used antibiotics (87.2%). Many patients were additionally treated with other antibiotic‐like aminoglycosides (3.7%) or with antibiotics that can cover methicillin‐resistant staphylococcus aureus (20.2%) or *Pseudomonas aeruginosa* (35.8%). Oseltamivir was occasionally used (5.5%) for the treatment of influenza. Chest physiotherapy was the most frequently used (57.8%) adjunctive therapy; 33.9% of patients were intubated and underwent invasive mechanical ventilation. After treatment, 81.7% of patients had improvements on chest radiography.

**TABLE 2:** Characteristics of treatments for preoperative pneumonia

Characteristics	Number (%)
Initial antibiotics^†^	
Fluroquinolones/beta‐lactams^‡^	95 (87.2)
Aminoglycosides^§^	4 (3.7)
Coverage for MRSA^¶^	22 (20.2)
Coverage for *Pseudomonas aeruginosa* ^||^	39 (35.8)
Antiviral therapy	
Oseltamivir	6 (5.5)
Adjunctive therapy	
Chest physiotherapy	63 (57.8)
Noninvasive mechanical ventilation	20 (18.3)
Invasive mechanical ventilation	37 (33.9)
Corticosteroid	11 (10.1)
Immunoglobin	4 (3.7)
Improvements on chest radiography before surgery	89 (81.7)

^†^ All diagnosed patients were treated with antibiotics.

^‡^ Fluroquinolone: levofloxacin, moxifloxacin; beta‐lactam: ceftriaxone, cefotaxime, cefoperazone etc.

^§^ Aminoglycoside: amikacin, gentamicin etc.

^¶^ Vancomycin or linezolid.

^||^ Papracillin‐tazobactam, cefepime, imipenem, meropenem etc.

Values are given as median (25th to 75th percentiles) or number (%).

MRSA, methicillin‐resistant staphylococcus aureus.

### *Outcomes of Patients with or without Preoperative Pneumonia*

The outcomes of patients are reported in Table 3; patients with preoperative pneumonia had longer hospital stays (15 *vs* 12 days, *P* < 0.001) and ICU length of stay (7 *vs* 0 days, *P* < 0.001) than patients free of preoperative pneumonia. Patients with preoperative pneumonia had higher 30‐day mortality rates (11.9% *vs* 5%, *P* = 0.002) and 1‐year mortality rates (33.9% *vs* 16.3%, *P* < 0.001).

**TABLE 3:** Comparison of outcomes between patient with or without preoperative pneumonia

Outcome	No preoperative pneumonia	Preoperative pneumonia	*P*
Hospital length stay (days)	12 (7–16)	15 (11–19)	<0.001
ICU length of stay (days)	0 (0–3)	7 (2–12)	<0.001
30‐day mortality	64 (5.0%)	13 (11.9%)	0.002
1‐year mortality	208 (16.3%)	37 (33.9%)	<0.001
Readmission within 1 year	474 (37.1%)	45 (41.3%)	0.379
Complications			
Surgical or implant related^†^	85 (6.6%)	10 (9.1%)	0.318
Deep venous thrombosis	19 (1.5%)	2 (1.8%)	0.776
Pulmonary embolism	11 (0.9%)	2 (1.8%)	0.312
Delirium	230 (18.0%)	13 (11.9%)	0.101
Postoperative pneumonia^‡^	53 (4.2%)	8 (7.3%)	0.183
Urinary tract infection	27 (2.1%)	3 (2.8%)	0.660
Acute myocardial infraction	6 (0.5%)	2 (1.8%)	0.071
Acute heart failure	43 (3.4%)	8 (7.3%)	0.034
Acute kidney injury	23 (1.8%)	6 (5.5%)	0.009

^†^ Periprosthetic fracture, hip instability or dislocation, implant failure, surgical site infection etc.

^‡^ New airspace opacity, lobar consolidation, or interstitial opacities on chest radiograph and symptoms and signs of an acute lower respiratory tract infection.

Values are given as median (25th to 75th percentiles) or number (%)

ICU, intensive care unit.

### *Risk Factors of 1‐year Mortality in Patients with or without Preoperative Pneumonia*

An analysis of risk factors for 1‐year mortality is reported in Table 4. Based on previous studies, risk factors that may be associated with 1‐year mortality (including age, BMI, ADL score, surgical delay, anesthesia type, ASA score, preoperative pneumonia and other comorbidities, and laboratory test results) were analyzed by univariate and multivariate analysis. After processing, the factors remained in the model, including age (≥84 years, *OR* 1.46, 95% *CI* 1.08–1.60, *P* = 0.027), BMI (<18.5 kg/m^2^, *OR* 2.23, 95% *CI* 1.52–3.17, *P* < 0.001), anesthesia type (regional, 0.87, 95% *CI* 0.19–0.97, *P* = 0.042), preoperative pneumonia (*OR* 1.45, 95% *CI* 1.39–3.52, *P* = 0.002), congestive heart failure (*OR* 2.05, 95% *CI* 1.57–6.21, *P* < 0.001), and chronic kidney disease (*OR* 1.73, 95% *CI* 1.50–2.62, *P* < 0.001). A subgroup analysis of 1‐year mortality grouped by aforementioned risk factors was performed (Table 5), with patients of older age (22.6% *vs* 17.3%, *P* = 0.025), with lower BMI (18.9% *vs* 12.2%, *P* = 0.002), with congestive heart failure (23.5% *vs* 17.1%, *P* = 0.030), with chronic kidney disease (21.8% *vs* 12.5%, *P* = 0.021), and who received general anesthesia (19.2% *vs* 14.4%, *P* = 0.047) having a higher rate of 1‐year mortality.

**TABLE 4:** Risk factors of 1‐year mortality in patients with or without preoperative pneumonia

	Univariate analysis			Multivariate analysis		
Variables	*OR*	95% *CI*	*P*	*OR*	95% *CI*	*P*
Age						
≥84	1			1		
<84	1.69	1.02–1.77	0.025	1.46	1.08–1.60	0.027
BMI (kg/m^2^)						
≥18.5	1					
<18.5	2.59	1.95–3.81	<0.001	2.23	1.60–3.18	0.033
ADL score						
≥45	1					
<45	1.98	0.67–2.87	0.219			
Surgical delay (h)						
≤24	1					
>24	1.33	0.39–2.51	0.650			
Anesthesia type						
General	1					
Regional	0.74	0.43–0.83	<0.001	0.87	0.19–0.95	0.042
ASA score						
≤3	1					
>3	0.80	0.36–1.76	0.581			
WBC count (×10^9^/L)						
≤10	1					
>10	0.77	0.42–1.11	0.150			
C‐reactive protein (mg/L)						
≤200	1					
>200	1.73	0.87–4.32	0.813			
Preoperative pneumonia						
No	1			1		
Yes	1.78	1.28–5.16	<0.001	1.45	1.39–3.52	0.021
Congestive heart failure						
No	1			1		
Yes	2.46	1.43–5.87	<0.001	2.05	1.52–3.21	<0.001
Prior myocardial infarction						
No	1					
Yes	1.30	0.82–2.04	0.250			
COPD						
No	1					
Yes	1.40	0.89–2.15	0.131			
Stroke						
No	1					
Yes	0.88	0.35–1.61	0.983			
Dementia						
No	1					
Yes	0.91	0.74–1.22	0.754			
Diabetes						
No	1					
Yes	1.16	1.05–1.23	0.023			
Chronic kidney disease						
No	1			1		
Yes	1.25	1.01–2.33	0.018	1.73	1.50–2.62	<0.001

ADL, activities of daily living; ASA, The American Society of Anesthesiologists; BMI, body mass index; CI, confidence interval; CURB‐65, confusion, uremia, respiratory rate, blood pressure, age ≥65 years; OR, odds ratio; WBC, white blood cell count.

**TABLE 5:** Subgroup analysis of 1‐year mortality in patients with or without preoperative pneumonia

Variables	1‐year mortality (%)	*P*
Age		0.025
≥84	22.6	
<84	17.3	
BMI (kg/m^2^)		0.002
≥18.5	12.2	
<18.5	18.9	
Anesthesia type		0.047
General	19.2	
Regional	14.4	
Congestive heart failure		0.030
No	17.1	
Yes	23.5	
Chronic kidney disease		0.021
No	12.5	
Yes	21.8	

BMI, body mass index.

### *Distribution of Preoperative Pneumonia Severity and Its Influences on 1‐year Mortality*

The distribution of patients with preoperative pneumonia in different severity groups and the adjusted (age, BMI, anesthesia type, congestive heart failure, and chronic kidney disease) odd ratios (*OR*) of 1‐year mortality with each CURB‐65 score are depicted in Table 6. The 1‐year mortality of these patients ranged from 18.2% to 81.8%. The *P‐*value for the trend of CURB‐65 scores to 1‐year mortality after adjustment for BMI and anesthesia type was 0.006. When CURB‐65 ≥3, patients had significant risk of 1‐year mortality. Patients with a score of 5 had the highest 1‐year mortality of 81.8% (*OR* = 2.87, 95% *CI* 2.10–7.69); comparing the rate of 1‐year readmission, there were no difference (41.3% *vs* 37.1%, *P* = 0.379).

**Table 6:** Adjusted OR of observed 1‐year mortality stratified by CURB‐65 scores

CURB‐65 score	Observed 1‐year mortality % (death/total)	*OR* (95% *CI*)	*P*	*P* for trend
0	18.2 (2/11)	1 (As reference)		0.006
1	20.7 (6/29)	1.17 (0.20–6.94)	0.860	
2	28.6 (8/28)	1.80 (0.32–10.23)	0.507	
3	38.5 (6/13)	3.85 (1.08–25.30)	0.047	
4	41.2 (8/17)	4.00 (1.66–24.30)	0.042	
5	81.8 (7/11)	7.87 (2.10–38.12)	0.039	

Odds ratio (*OR*) analyzed by multivariate logistic regression with adjustments for age, BMI, anesthesia type, congestive heart failure and chronic kidney disease. CURB‐65, confusion, uremia, respiratory rate, blood pressure, and age ≥65 years.

### *Survival Analysis of Patients with Preoperative Pneumonia*

**Fig. 1:**
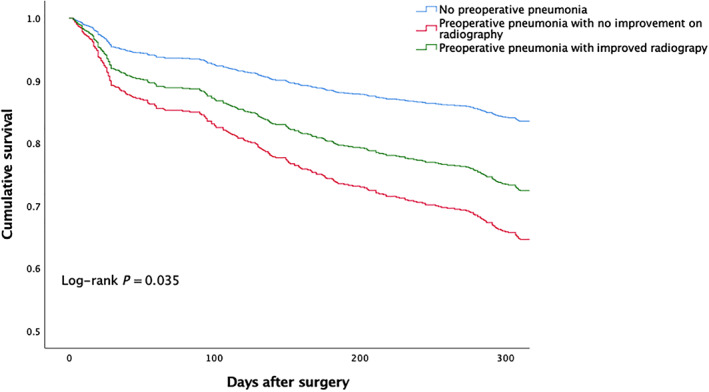
Survival curves of patients with or without preoperative pneumonia. Adjusted for baseline difference between groups (ADL score, WBC, and C‐reactive protein) and possible risk factors of 1‐year mortality (age, BMI, anesthesia type, congestive heart failure and chronic kidney disease). ADL, activities of daily living; BMI, body mass index; WBC, white blood cell count.

The survival curves of patients free of preoperative pneumonia and patients with preoperative pneumonia (improved or unimproved chest radiography) are depicted in Fig. 1. The analysis was performed using a Cox regression model, which adjusted for baseline differences between groups (ADL score, WBC, and C‐reactive protein) and possible risk factors of 1‐year mortality (age, BMI, anesthesia type, congestive heart failure and, chronic kidney disease). When compared to patients without preoperative pneumonia, patients with preoperative pneumonia (improved or unimproved) had significantly lower probability of survival; patients with unimproved preoperative pneumonia had the lowest survival rate over time (log‐rank, *P* = 0.035).

**Fig. 2:**
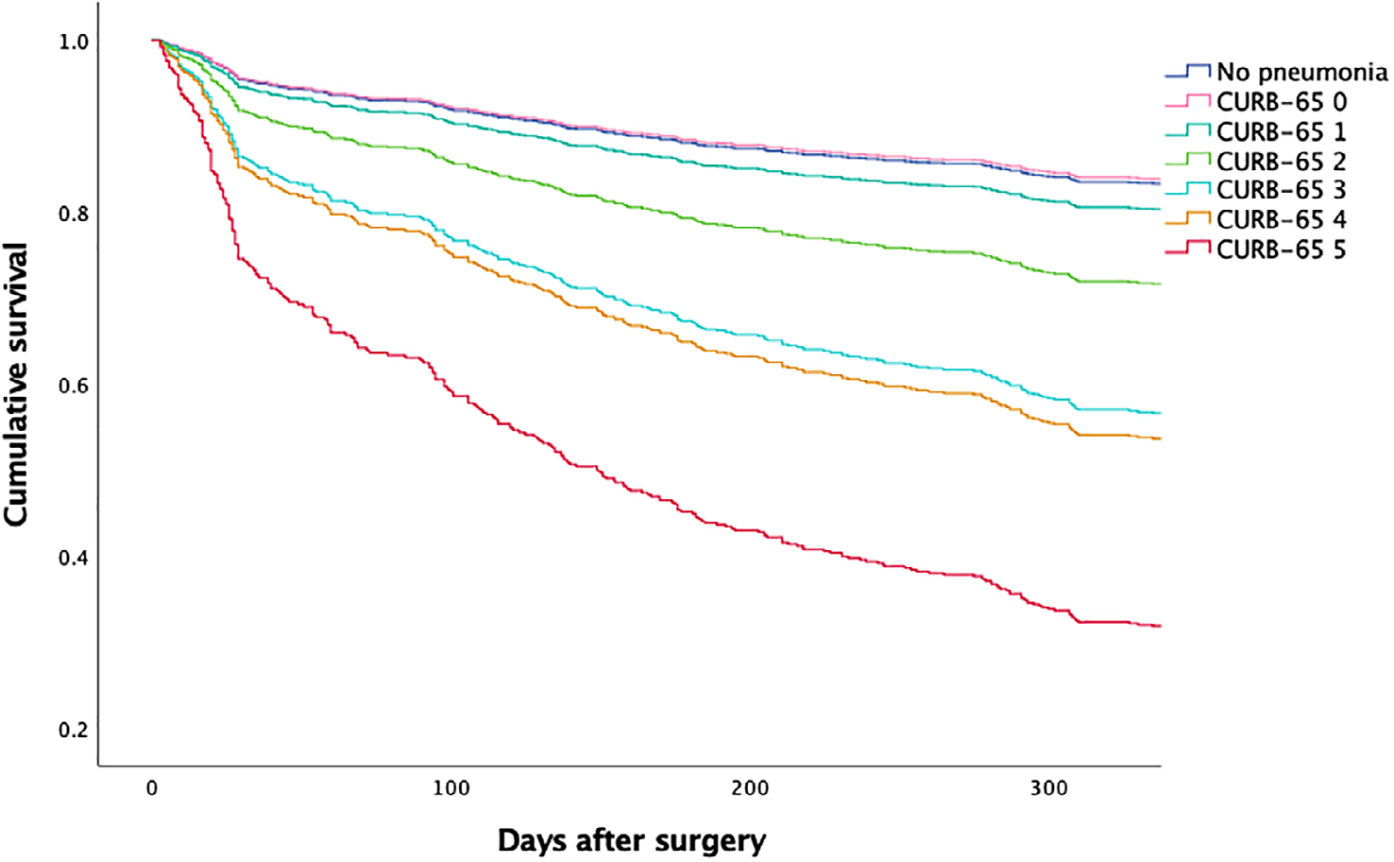
Survival curves of patients without preoperative pneumonia or patients with different severity of preoperative pneumonia. HR of mortality in patients with different CURB‐65 scores to patients without preoperative pneumonia: CURB‐65 0 (*HR* 0.96, 95% *CI* 0.24–3.86, *P* = 0.958), 1 (*HR* 1.72, 95% *CI* 0.88–3.35, *P* = 0.113), 2 (*HR* 2.96, 95% *CI* 0.93–5.21, *P* = 0.067), 3 (*HR* 6.28, 95% *CI* 2.95–13.35, *P* < 0.001), 4 (*HR* 3.41, 95% *CI* 1.69–6.92, *P* = 0.001), and 5 (*HR* 6.28, 95% *CI* 2.95–13.35, *P* < 0.001). Adjusted for baseline difference between groups (ADL score, WBC, and C‐reactive protein) and possible risk factors of 1‐year mortality (age, BMI, anesthesia type, congestive heart failure, and chronic kidney disease). ADL, activities of daily living; BMI, body mass index; CURB‐65, confusion, uremia, respiratory rate, blood pressure, age ≥65 years; *CI*, confidence interval; *HR*, hazard ratio; WBC, white blood cell count.

Analysis of the survival condition of patients without preoperative pneumonia or with different pneumonia severity (evaluated with CURB‐65) was also performed using COX regression, adjusting for the aforementioned factors (Fig. 2). Only patients with a CURB‐65 score of 3 (*HR* 3.12, 95% *CI* 1.39–7.03, *P* = 0.006), 4 (*HR* 3.41, 95%*CI* 1.69–6.92, *P* = 0.001), or 5 (*HR* 6.28, 95% *CI* 2.95–13.35, *P* < 0.001) had significant risk of 1‐year mortality when compared with patients that were free of preoperative pneumonia.

### *Complications of Patients with or without Preoperative Pneumonia*

Incidence of acute heart failure (7.3% *vs* 3.4%, *P* = 0.034) and acute kidney injury (5.5% *vs* 1.8%, *P* = 0.009) were higher in patients with preoperative pneumonia, while no difference in incidence was observed for other complications, including surgical or implant‐related deep venous thrombosis or pulmonary embolism (Table 3).

## Discussion

In this cohort of geriatric patients undergoing hip fracture surgery or arthroplasty, patients with preoperative pneumonia had higher 1‐year mortality and worse outcomes than patients without this condition. In addition, when evaluating the severity of pneumonia using CURB‐65, patients with a score over 3 had higher risk of 1‐year mortality.

The incidence of preoperative pneumonia in our cohort was higher (7.86%) than in previous studies (1%–3.2%)^20^, ^21^, ^22^. A possible cause for this phenomenon was that we applied a case‐by‐case reviewing method for the diagnosis of preoperative pneumonia, which may achieve more positive diagnoses for cases with mild symptoms than using a data extraction method and medical records or a social health database.

### *Clinical Outcomes of Patients with or without Preoperative Pneumonia*

In our cohort, outcomes of patients with preoperative pneumonia were generally worse than for patients without this condition (Table 3). In addition, among other baseline conditions and various comorbidities, preoperative pneumonia was identified as an independent risk factor of 1‐year mortality after surgery (Table 4). We also observed a high ICU admission rate in these patients, which can be explained by high possibility of status aggravation after surgery of patients with preoperative pneumonia. These patients also had higher rates of acute heart failure and acute kidney injury (AKI). As reported in previous studies^27^, ^28^, ^29^, community‐acquired pneumonia substantially increases the risk of heart failure and AKI in various populations; the occurrence of these two complications may result in the high 1‐year mortality rate of patients with preoperative pneumonia.

### *Patient Response to Pneumonia Treatment and its Influence on Survival*

As depicted in Fig. 1, the survival rate of patients with preoperative pneumonia was decreasing over time and was significantly lower than for those without the condition. Moreover, despite use of antibiotics and adjunctive therapies, many patients may not have improvement on chest radiographies (Table 2); the survival rate was lowest in these patients (Fig. 1). Although the monitoring of chest X‐rays had no additional value in predicting patients’ clinical course in a study focused on community‐acquired pneumonia^30^, our finding indicates that patients’ response to pneumonia‐related treatment may be useful in predicting prognosis in this very population.

### *CURB‐65 Score as a Prediction Tool of Long‐term Survival*

As a prediction tool for prognosis of patients with pneumonia, CURB‐65 has been validated as a valuable scoring system for both 30‐day and 1‐year mortality in various populations^24^, ^25^, ^31^, ^32^. Barlow *et al*. reported that patients that were diagnosed with community‐acquired pneumonia and had low CURB‐65 scores (i.e., 0–1) did not differ much in 30‐day mortality, which was similar to our findings^33^. Other studies have shown that a cut‐off value of 3 for CURB‐65 had the strongest prediction value of mortality in these patients^24^, ^34^. Our findings indicate that in patients admitted for hip fracture surgery or arthroplasty, CURB‐65 ≥3 indicated significant increased risk of 1‐year mortality in patients with preoperative pneumonia. In addition, patients with a CURB‐65 ≤2 did not differ much in survival status, even though there was a linear correlation between CURB‐65 and 1‐year mortality when CURB‐65 ≥3 (Table 4). Whether the integration of severity evaluation with CURB‐65 and different therapeutic strategies can improve outcomes is yet to be tested in further studies.

### *Limitations*

This study must be interpreted in the context of limitations. First, the nature of the retrospective and single center study resulted in limited power to rule out potential confounding factors of the prognosis of patients. Second, we were not able to include functional analysis for patients enrolled in this study because of an inadequate response from patients in the follow‐up period. This compromised the evaluation of influences that preoperative pneumonia had on patients. These aspects need to be evaluated in more methodologically rigorous studies in the future.

## Conclusion

In this single center retrospective study, preoperative pneumonia was identified as an independent risk factor of 1‐year mortality in geriatric patients undergoing hip fracture surgery or arthroplasty, with a CURB‐65 score ≥3 indicating a higher risk of mortality.
